# Meningococcal Vaccination in High-Risk Patients: A Systematic Approach to Evaluating Coverage and Patient Catch-Up Through Healthcare Databases

**DOI:** 10.3390/vaccines13030287

**Published:** 2025-03-08

**Authors:** Rafael Ruiz-Montero, Álvaro Serrano-Ortiz, Mario Rivera-Izquierdo, Piedad Galvache Murillo-Rico, Ana Moñiz-Díez, María Ángeles Onieva-García, Eloy Girela-López, Inmaculada Salcedo-Leal

**Affiliations:** 1 Preventive Medicine and Public Health Unit, Reina Sofía University Hospital, 14004 Córdoba, Spain; rafael.ruiz@uco.es (R.R.-M.); mariaa.onieva.sspa@juntadeandalucia.es (M.Á.O.-G.); minmaculada.salcedo.sspa@juntadeandalucia.es (I.S.-L.); 2 Department of Medical and Surgical Sciences, University of Córdoba, 14071 Córdoba, Spain; piedadgalvache@gmail.com; 3 Preventive Medicine and Public Health Research Group, Maimonides Biomedical Research Institute of Córdoba (IMIBIC), 14004 Córdoba, Spain; alvaro.serrano.sspa@juntadeandalucia.es (Á.S.-O.); ana.moniz@imibic.org (A.M.-D.); 4 Preventive Medicine and Public Health Unit, Healthcare Management Area South of Córdoba, 14940 Cabra, Córdoba, Spain; 5 Department of Preventive Medicine and Public Health, University of Granada, 18016 Granada, Spain; 6 Instituto de Investigación Biosanitaria de Granada (ibs.GRANADA), 18014 Granada, Spain; 7 Centros de Investigación Biomédica en Red de Epidemiología y Salud Pública (CIBERESP), 28029 Madrid, Spain; 8 Section of Legal and Forensic Medicine, Faculty of Medicine and Nursing, University of Córdoba, 14004 Córdoba, Spain; eloygirela@uco.es

**Keywords:** meningococcal infections, *Neisseria meningitidis*, vaccination, vaccination coverage, immunization programs

## Abstract

**Background**: Invasive meningococcal disease (IMD) can lead to severe and fatal outcomes. Vaccines against meningococcus (serogroups B, MenB; or ACWY, MenACWY) are recommended for patients at high risk of developing IMD. Our aim was to identify high-risk patients through a systematic search of medical codes and to evaluate vaccination coverage by high-risk group. **Methods**: An observational retrospective study was conducted in patients discharged at Reina Sofía University Hospital (Cordoba, Spain) from 1 January 2000, to 31 December 2023. Selection of high-risk patients was conducted through national administrative and clinical databases and vaccination coverage was determined through the Andalusian electronic vaccine database. Vaccine coverages of MenB and MenACWY were calculated within risk groups. Finally, bivariate analyses were conducted to assess the potential association between coverage, sex, and the year of admission. **Results**: A total of 2689 patients with 2710 high-risk conditions for IMD were identified from the databases searched. Of the 1755 requiring MenB vaccination, only 624 (35.6%) had received one dose and 558 (31.8%) two doses. Of the 2710 requiring MenACWY vaccination, only 784 (28.9%) had received one dose and 520 (19.2%) two doses. Patients with properdin-complement deficiencies showed the lowest vaccination rates (<10%). For the rest of the high-risk groups, vaccination coverages were significantly lower when the condition was diagnosed before the immunization guideline recommendations (*p* < 0.001). **Conclusions**: The identification of high-risk patients through databases using R-coded algorithms is both feasible and effective for identifying and catching-up patients for vaccination. The population at risk of IMD lacks adequate meningococcal vaccination coverage. Our methodology can serve to identify patients in other regions and for different vaccines.

## 1. Introduction

Invasive meningococcal disease (IMD) is a severe and often life-threatening condition caused by *Neisseria meningitidis* (meningococcus) [1]. Meningitis and septicemia represent the most common clinical manifestations within the spectrum of IMD and have a case fatality rate of 10–15% [2]. *N. meningitidis* is an obligate human pathogen and meningococcal transmission occurs through close contact with infected upper respiratory tract droplets or secretions [2]. Asymptomatic nasopharyngeal carriage prevalence is about 10% in adults [2]. There are 13 identified serogroups of *N. meningitidis*, 6 of which (A, B, C, W, X, and Y) are responsible for the majority of IMD worldwide [3].

In developed regions, the incidence of endemic IMD has been low in recent decades, with overall rates ranging from 0.3 to over 3.0 cases per 100,000 population, predominantly involving serogroups C and B [4]. In recent years, IMD incidence in Spain has fluctuated, with a declining trend until 2014, a subsequent rise peaking in 2018–2019, and a sharp drop during the COVID-19 pandemic. However, since 2022, an upward trend has been observed, approaching pre-pandemic levels (0.83 in 2019 vs. 0.68 in 2024) [5,6]. Serogroups B, W, Y, and C were the most common, with serogroup B being markedly more prevalent than the others. Specifically, in Andalusia (a region of southern Spain with over 8.5 million inhabitants), IMD incidence in 2023 was 0.5 cases per 100,000 inhabitants, comparable to national incidence [6].

This progressive reduction is, at least partially, attributable to immunization programs, which play a key role in disease prevention [7,8]. Currently, effective vaccines are available, including the meningococcal C conjugate vaccine, the multivalent conjugate vaccine targeting serogroups A, C, W, and Y (MenACWY), and recombinant protein-based vaccines against serogroup B (MenB) [7,8]. Vaccines available in Spain against MenB and MenACWY are presented as Appendix A (Table A1). Despite the fact that the definition of individuals at higher risk for IMD (HR-IMD) is not fully standardized [7], the Andalusian Public Health System, according to Spanish Ministry of Health recommendations, includes as high-risk patients those with asplenia or conditions affecting the spleen, certain disorders of the complement system, previous IMD, outbreak, laboratory personnel, hematopoietic stem cell transplantation (HSCT), people living with HIV (PLWH), and those receiving complement inhibitor treatments (e.g., eculizumab or ravulizumab), among others [9,10].

In Spain, clinical physicians identify high-risk patients eligible for meningococcal vaccination and subsequently refer them to the Preventive Medicine and Public Health units of their designated hospitals. Within these units, a personalized vaccination schedule is developed, with the option for patients to complete the schedule either in the same unit or at their primary care center. This patient selection system (which is also the main approach in other healthcare systems) operates sub-optimally for several reasons: excessive healthcare workload, limited consultation time, interoperability issues between applications and healthcare systems, and insufficient coordination between primary and hospital care. Additionally, population mobility, irregular healthcare attendance, unequal literacy, and outdated vaccination records further hinder effective patient recall [11].

The identification of individuals who may benefit from vaccination through the integration of coded data from health databases has the potential to not only enhance the efficiency of resource management but also improve the quality of care, decrease health inequities, streamline processes, and facilitate evidence-based decision-making. The need to prioritize high-risk patients for SARS-CoV-2 vaccination encouraged the use of healthcare databases for their identification. However, this methodology has not been standardized or applied to other vaccines or risk groups. To date, there is no institutional program available for this purpose and, to the best of our knowledge, there are no published articles with feasibly applicable methodologies for this purpose that are not related to SARS-CoV-2 vaccines [12].

The Andalusian Public System of Health employs Information and Communication Technologies for the integration of information pertaining to each citizen into a single health record, identified by a Unique Health Record Number (NUHSA).

Diraya is an integrated management and information system for healthcare, which the Andalusian Public System of Health utilizes in its regional health service. All vaccinations are systematically registered in Diraya Vaccines. Additionally, hospitals in Spain are required to complete a Minimum Basic Data Set (CMBD) upon patient discharge, in which diseases, procedures, and treatments received during hospitalization are coded. This hospital discharge database is a standard feature in most European Union countries. In Andalusia, the CMBD is compiled following the International Classification of Diseases (ICD), encompassing procedures and diagnoses according to ICD-9 and ICD-10 classifications [13]. Consequently, electronic data can be accessed to identify patients with HR-IMD conditions and assess their vaccination status. This enables the evaluation of vaccine coverage and facilitates accurate and efficient identification and catch-up for vaccination.

Moreover, after the COVID-19 pandemic, a widespread decline in vaccination coverage has been observed, particularly affecting the most vulnerable populations. In response, health authorities in several countries are implementing new recruitment strategies to enhance adherence to vaccination programs [14].

The aim of this study was to identify all patients with HR-IMD conditions discharged from Reina Sofía University Hospital from 1 January 2000, to 31 December 2023, and to quantify vaccine coverage in this population stratified by high-risk group.

## 2. Materials and Methods

The results of this study were reported according to the Strengthening the Reporting of Observational Studies in Epidemiology (STROBE) guidelines [15].

### 2.1. Study Design and Setting

This study was designed as an observational retrospective study. The target population included all patients with HR-IMD conditions discharged from the Reina Sofía University Hospital of Cordoba, Spain, from 1 January 2000, to 31 December 2023. Our center is a tertiary care hospital with 1000 beds and serves as a reference hospital for the province of Cordoba (>750,000 inhabitants) and Jaen (>600,000 inhabitants) for highly complex processes. Its reference population for the first level of care is 450,000 inhabitants.

The sample was composed of patients with HR-IMD conditions who remain active (alive) in the User Database (BDU) as of 15 March 2024 (end of retrospective follow-up). Reasons for being passive are detailed in the study variables section. Since the specific recommendations for immunization schedules may vary among HR-IMD conditions, individuals with several HR-IMD factors may or may not have been correctly or timely vaccinated, depending on the comorbidity considered. For this reason, we decided to analyze the HR-IMD conditions of each patient as separate study units. Inclusion criteria of HR-IMD conditions were selected according to the International Classification of Diseases, ninth and tenth revision (ICD-9 and ICD-10) [13] (Table 1), according to the coding selected for such risk groups. Data on vaccines were updated to 15 March 2024, the date on which the cross-referencing of data from the health databases was performed.

### 2.2. Data Sources and Study Variables

The data sources used for this study were the CMBD, the BDU, and Diraya Vaccines. The CMBD included a register of patients discharged from the Reina Sofía University Hospital. The variables are standardized at the national level and include administrative, clinical, and surgical data. The BDU is a database that includes the basic sociodemographic data of patients in Andalusia and their administrative healthcare status within the Health System. The Diraya Vaccines is the regional registry of vaccinations in Andalusia.

The following sequence of patient selection was used to determine the cases of HR-IMD conditions and identify the sample: (1) Identification of HR-IMD groups eligible for meningococcal vaccination according to the Andalusian Vaccination Program against Meningococcal Disease 2023 [10]. These groups were asplenia, properdin-complement deficiency, previous IMD, PLWH, laboratory personnel, and patients involved in an outbreak, since April 2014, according to the immunization guideline of the Andalusian Ministry of Health at this date. In July 2018, according to an updated immunization guideline, a new high-risk group was included: HSCT recipients. (2) Search and collection of ICD-9 and ICD-10 codes (diagnoses and procedures) related to the HR-IMD groups. This search was conducted in the electronic edition of the Spanish Ministry’s International Classification of Diseases ICD-9 and ICD-10 [13]. (3) Extraction of data of the previously defined codes of HR-IMD groups that were discharged from our center during the study period. These data were requested and collected from the CMBD register. (4) Application of the inclusion and exclusion criteria to the list of patients admitted to the CMBD register. (5) Selection of the first episode in which the patient was diagnosed with the HR-IMD condition and removal of subsequent duplicated data. (6) Linking of active patients with their vaccination records through the NUHSA individual numbers for matching databases (CMBD and Diraya Vaccines) using R. (7) Assessing the specific vaccination schedule per patient and risk group. We used our regional immunization guidelines [10] to assess whether patients were correctly vaccinated against meningococcus on the selected date (15 March 2024).

For the evaluation of vaccination status, the following variables were considered: risk condition, number of doses administered per vaccine, and dates of each dose. Based on these criteria, patients were classified according to the action required by the healthcare system based on their vaccination status (see Table A1 for the specific recommendations): (a) correctly vaccinated: patients who have fully completed the recommended vaccination schedule or those for whom the minimum recommended interval between doses has not been exceeded, (b) vaccination initiated but pending scheduling: patients who have initiated vaccination but the recommended interval between doses has been exceeded and who therefore need to be scheduled for the next dose; or (c) vaccination not initiated: patients who need an appointment to initiate the vaccination schedule.

The variables included in the study were:

• Status: active or passive
• Reason for passive status: dead at the end of follow-up (15 March 2024), not having NUHSA (unique health identifier for Andalusia), living in another autonomous community, and other administrative problems with the Andalusian Health Service. Date of discharge from the first intervention or diagnosis that led to the inclusion in the HR-IMD group.
• Time where the risk condition for vaccine recommendation first appeared: before or after the regional immunization guidelines for meningococcal vaccination.
• Sex: male or female
• Age at the time of data matching.
• Risk group (Table 1).
• ICD-9 and ICD-10 codes.
• Name of the vaccine required: Bexsero^®^, Trumenba^®^, Nimenrix^®^, MenQuadfi^®^, or Menveo^®^.
• Date of vaccine administration.
• Number of doses received of each meningococcal vaccine.

### 2.3. Statistical Analysis of Data

All analyses were conducted separately for each HR-IMD group. A descriptive study of the variables was conducted using absolute and relative frequencies for qualitative variables. In the case of quantitative data, a normality test was performed prior to calculating measures of central position and dispersion. Where variables showed normal distribution, we calculated the arithmetic mean, standard deviation, and minimum and maximum values. Otherwise, we obtained median values and percentiles.

Subsequently, a bivariate analysis was conducted separately for each high-risk group. To analyze potential differences of subgroups according to sex, age, and the moment of presentation of the high-risk condition (before or after the immunization guidelines), statistical tests were applied. Chi-square tests for qualitative variables were performed when the conditions of applications were met; otherwise, Fisher exact tests were conducted. For age groups, Mann–Whitney and homoscedasticity tests were applied. The data were collected, processed, and analyzed using R statistical software (R Core Team, Vienna, Austria), version 4.4.

### 2.4. Ethical Considerations

The study was conducted in accordance with the Declaration of Helsinki, and the protocol was approved by the Research Ethics Committee of Cordoba, Spain (Project Ref. No. 5718, approved on 28 November 2023).

## 3. Results

### 3.1. Selection of the Sample

During the 24 years of the study period (1 January 2000, to 31 December 2023), a total of 970,302 patients were discharged from our hospital.

**Figure 1:**
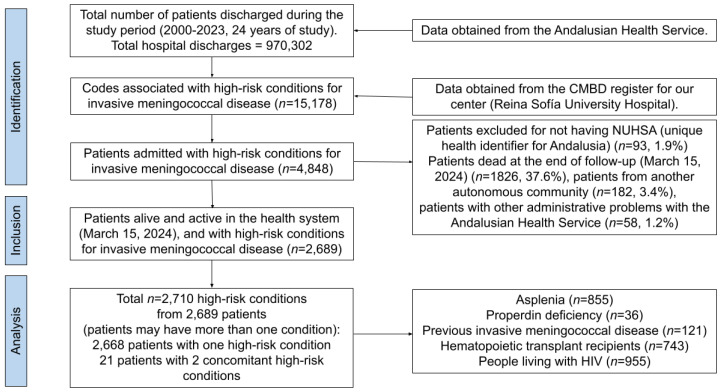
Flow chart of the sample selection.

According to HR-IMD conditions, 40 codes were identified in ICD-9 Diagnostics, 11 in ICD-9 Procedures, 89 in ICD-10 Diagnostics, and 73 in ICD-10 Procedures, as summarized in Table 1. Briefly, 15,178 codes were identified corresponding to 4848 patients. After applying the inclusion and exclusion criteria, the sample was composed of 2689 patients presenting 2710 high-risk conditions (as 21 patients met several high-risk conditions). Details on the flow chart of the study selection process are presented as Figure 1.

### 3.2. Characteristics of the Sample

**Figure 2:**
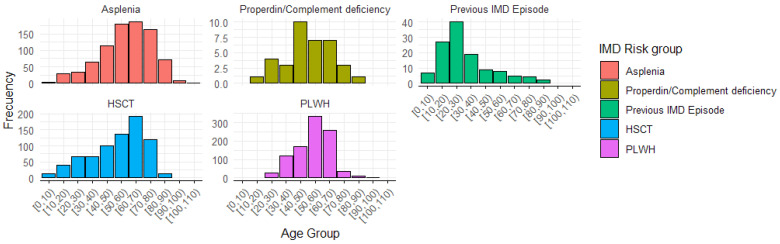
Age distribution by high-risk condition for invasive meningococcal disease.

The mean age of the patients included in the study was 53.8 (standard deviation = 17.1), and 1073 (40.0%) were females. A total of 2668 (99.2%) patients presented a single HR-IMD condition, whilst 21 (0.8%) patients showed two HR-IMD conditions. Table 1 shows the different HR-IMD groups and their corresponding ICD codes. As no patient was identified in the outbreak and laboratory personnel groups, six HR-IMD groups were finally identified (asplenia, properdin deficiency, previous IMD, hematopoietic stem cell transplantation, complement inhibitor therapies, and people living with HIV). Table 2 shows the distribution of patients by HR-IMD group, age, and sex. Briefly, the median age of all HR-IMD conditions was around 55 to 65 years for males and females, except for previous IMD (around 26 years old). Figure 2 shows the distribution of age groups of each HR-IMD condition.

Normality tests for age by sex were <0.05 for all high-risk conditions (therefore, Mann–Whitney tests were performed) except for properdin-complement deficiency (*t*-test was performed), as shown in Table 2.

### 3.3. Number of MenB and MenACWY Doses Received by Each HR-IMD Condition

Regarding MenB, of the 1755 candidates that required vaccination, 1131 (64.4%) had received zero doses of vaccine (did not initiate vaccination schedule). Data stratified by HD-IMD condition ranged from 60.4% in patients with asplenia to 94.4% in patients with properdin deficiency. Regarding MenACWY, of the 2710 candidates that required vaccination, 1926 (71.1%) had received zero doses of vaccine, ranging from 60.7% in patients with hematopoietic stem cell transplantation to 91.7% in patients with properdin deficiency. The detailed numbers of MenB and MenACWY doses by HR-IMD condition are presented in Table 3. Regarding differences by sex, females showed higher vaccination rates with MenB (*p* < 0.001) and MenACWY (*p* < 0.001) in the group of asplenia and lower vaccination rates with MenACWY in the group of PLWH (*p* < 0.001).

### 3.4. Vaccination Status at the Time of the Analysis

The MenB and MenACWY status at the time of the analysis (15 March 2024) and the required recommendations are summarized in Table 4. A total of 3082 conditions were identified for citation to initiate vaccination schedule, with no doses received: 1145 (64.7%) for MenB and 1937 (71.1%) for MenACWY. Furthermore, another 721 patients were identified for citation to continue their vaccination schedule according to the regional immunization guidelines, 493 (42.1%) for MenB and 172 (6.3%) for MenACWY.

### 3.5. Vaccination Coverage by Start of the HR-IMD Condition

**Figure 3:**
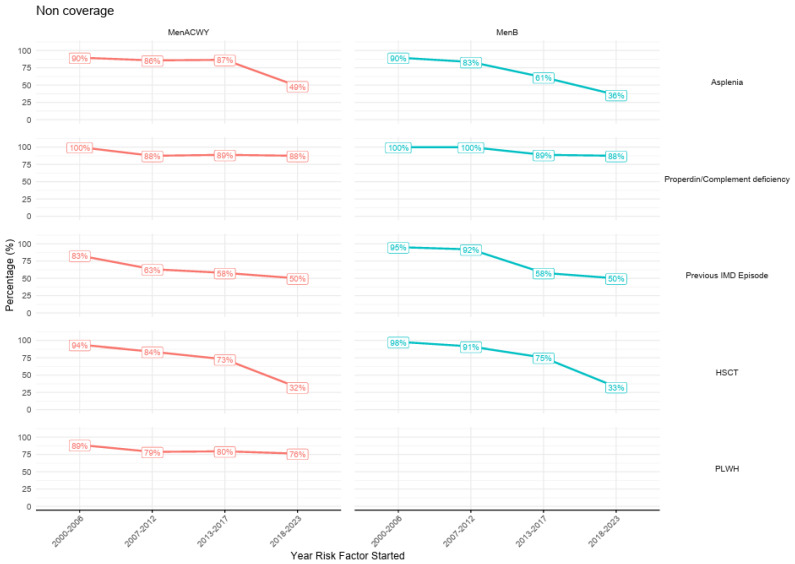
Vaccine non-coverage by risk group based on the date of diagnosis of the high-risk condition.

Figure 3 shows the MenB and MenACWY coverage by the start of the HR-IMD condition. It can be observed that patients who were diagnosed with HR-IMD conditions in earlier years (2000–2006) presented higher frequencies of non-coverage (89% to 100% for all HR-IMD conditions and both vaccines) that those starting in more recent years (2017–2023), especially for asplenia (36% of non-coverage for MenB and 49% for MenACWY) and hematopoietic stem cell transplantation recipients (33% of non-coverage for MenB and 32% for MenACWY). Table 5 shows the coverage of meningococcal vaccines according to the time of HR-IMD condition, whether it was diagnosed before or after the introduction of regional immunization guidelines. For all risk groups, the coverage of both MenB and MenACWY was considerably lower when the HR-IMD condition was diagnosed before the immunization guideline, except for the group of properdin-complement deficiencies, which showed high percentages of non-coverage regardless of time of diagnosis.

## 4. Discussion

This study assessed meningococcal vaccination status across various high-risk groups for invasive meningococcal disease in a southern Spanish population, employing a systematic, comprehensive methodology for patient identification via ICD codes. Concerningly, a high proportion of eligible individuals did not initiate the immunization schedule: 64.4% of the candidates for MenB vaccination and 71.1% of the candidates for MenACWY vaccination. Even fewer received at least two doses and thus achieved adequate immunization: 7.5% for MenB and 22.7% for MenACWY. These findings emphasize the critical need to address strategies that are truly effective in improving vaccination coverage among these patients.

Regarding asplenia, our study showed only a 39.6% coverage of MenB (37.1% of two doses) and 28.1% (20.7% of two doses) of MenACWY, data even worse for males. These figures align with those reported by Bianchi et al. in 2023 (30.9% of two-dose MenB and 37.7% of two-dose MenACWY) in splenectomized patients identified through ICD-9 codes. Other studies have reported vaccination coverages between 3.5% and 59% [16,17,18,19,20,21,22,23]. Given the increased risk of splenectomized patients to infections caused by encapsulated pathogens, it seems evident that recruitment of patients with asplenia for meningococcal vaccination is far from optimal worldwide. Properdin-complement-deficient patients showed the lowest vaccination initiation rates in our study (over 90% did not receive any dose), which is even higher than those reported in another study [24]. The rarity of this condition may contribute to clinician unawareness of vaccination recommendations, compounded by potential deficiencies in patient referral mechanisms. Prior IMD episodes represent another critical HR-IMD condition. Vaccination is crucial to prevent new infections, as a previous episode of IMD does not guarantee immunity against all serogroups. MenB vaccination is effective against serogroup B and may offer cross-protection against others. HSCT recipients are one of the groups with the highest degree of immunosuppression, so adequate immunization is highly relevant. Our patients showed 35–40% of vaccination initiation. Few studies have assessed meningococcal vaccine coverage in this group [25], although data from a pediatric population showed 47.7% of vaccine initiation and only 1.3% of vaccination adherence [26]. Finally, only 22% of PLWH received at least one dose of MenACWY, dropping to 16% among females. Previous studies in PLWH have reported only a 3% coverage in Germany [27], 10.8% (two doses) in the US [28], and around 50% in Australia [29]. We could not identify any patients under the HR-IMD conditions “Outbreak” or “Laboratory personnel” as no codes matched these categories. This highlights the crucial role of epidemiology and public health services in implementing outbreak mitigation and control measures, including vaccinating close contacts of cases. Likewise, occupational health and workplace safety programs are responsible for identifying at-risk laboratory personnel and ensuring appropriate meningococcal vaccination. Algorithm-based detection from hospital codes appears inadequate for these groups, as well as for individuals receiving immunosuppressive treatments or complement inhibitors (i.e., eculizumab or ravulizumab). The use of Anatomical Therapeutic Chemical Classification System (ATC) codes to identify these patients within pharmacy databases should be considered; however, we were unable to achieve this in our study.

While a strikingly low proportion of patients across all HR-IMD conditions initiated the immunization schedule, the rates observed in those with properdin deficiency are particularly concerning. Conversely, patients with conditions requiring periodic monitoring by hospital physicians, intensive treatment, or surgical admission (i.e., asplenia, hematopoietic stem cell transplantation, and PLWH) exhibited higher (although not adequate) immunization initiation rates. This disparity underscores the importance of ensuring that healthcare providers are aware of immunization guidelines and have effective systems to facilitate patient referrals for vaccination.

Analysis of vaccination coverage across all HR-IMD conditions revealed significantly lower vaccination rates among individuals diagnosed before the implementation of current immunization guidelines, except for properdin-deficient patients. This suggests that patient catch-up following updated recommendations is ineffective. While current guidelines recommend a timely referral to immunization services following (and in some cases, preceding) HR-IMD diagnosis, individuals diagnosed prior to their implementation may not have been consistently identified and offered appropriate vaccination. Several factors likely contribute to this gap, including the following: (1) limited awareness of healthcare providers regarding evolving vaccination recommendations, (2) incomplete or outdated patient records (3) insufficient coordination between hospital physicians and public health services for patient identification and catch-up, and (4) a decline in follow-up visit frequency for certain HR-IMD conditions, hindering effective patient catch-up for immunization. These barriers collectively contribute to suboptimal vaccination rates and should be addressed through clinician education initiatives and improved patient catch-up systems using available databases. Furthermore, a lack of clear guidelines delineating responsibility for follow-up and immunization of these patients may contribute to suboptimal vaccination coverage [30]. A shared factor in all these considerations is logistical barriers. Shortage of healthcare personnel and overload of healthcare services complicate the catch-up of patients who are not newly diagnosed into the system, contributing to this phenomenon.

Various strategies have been explored to improve these rates. Health education for patients with asplenia or hyposplenia regarding the risk of infectious complications and preventive measures has been shown to enhance vaccination rates, as have active dissemination and reminder systems integrated into primary care. These interventions might be useful for other HR-IMD conditions [31,32]. However, the most effective approach to overcoming the previously identified challenges is the use of algorithms that automatically identify eligible patients through electronic health records and assess their immunization status via vaccination registries. To our knowledge, this study is among the most comprehensive in identifying HR-IMD conditions, using a thorough quantity of codes for their detection. Although occasional inaccuracies in patient coding within electronic health records are inevitable, these tools provide valuable clinical decision support, with errors being correctable upon review. Although early versions of such algorithms have proven effective for age-based vaccination recommendations, their accuracy in predicting immunization needs based on medical conditions remains limited [33]. More recently, a pilot study in southern Italy demonstrated the successful use of ICD and ATC codes within electronic health records for identifying high-risk patients eligible for vaccination when compared to diagnoses of general practitioners [34]. Our results reinforce the evidence that algorithms using robust and refined code listings, coupled with well-structured logical rules, can serve as powerful tools for patient identification and catch-up in vaccination programs.

The digitalization of health records in Andalusia has created a unique opportunity to enhance public health management. By developing and implementing an algorithm capable of analyzing data from electronic health records and vaccination registries, we have demonstrated the feasibility of proactively and automatically identifying patients who, based on their clinical and demographic profiles, are eligible for specific vaccinations but remain largely unvaccinated. Our study employs an innovative technique for patient identification, using ICD codification in electronic health records, a method also explored in previous studies [16]. Because of the importance of vaccination in high-risk groups, several initiatives have been conducted to improve their coverage, including attempts to catch up patients in specific health units or areas, or regional-level strategies targeting a specific risk group [17,35]. These approaches are often difficult to replicate in other areas because they rely heavily on a laborious manual process. Some authors have studied access to at-risk populations, although their approaches rely on the ability of professionals to know the recommendations and to recruit patients [36,37]. Some international experiences suggest that centralized immunization registries and electronic health records could be useful tools to increase vaccination coverage by sending digital reminders, especially to high-risk groups [27]. It should be noted, however, that there is no systematic approach to catch up with unvaccinated patients to date.

This study presents several limitations. First, although our data sources are highly reliable and cover a substantial proportion of the Andalusian population, patient identification is exclusively hospital-based. Most HR-IMD conditions require hospital care at some point, and therefore very few eligible patients are unidentified. However, conditions like complement inhibitors therapies or outbreaks are not always associated with hospital settings; therefore, to extend the catch-up of these patients it will be necessary to link hospital records with pharmacological and surveillance registries. Future incorporation of these data would help in including other at-risk groups (e.g., patients under immunosuppressive therapies, autoimmune diseases, etc.). Second, potential inaccuracies in medical coding could affect patient identification. However, as the CMBD relies on professional coding for diagnoses and hospital procedures, the impact of misclassification is expected to be minimal. Similarly, while some administered vaccines may not be registered in Diraya Vaccines, this limitation is likely marginal, given that this system has been in place since 2008 and vaccine registration is mandatory in both primary care and hospital settings.

The systematized approach tested in this study solves one of the major problems of vaccination in high-risk groups: defining the reference population, assessing its vaccination coverage, and facilitating patient catch-up for immunization. Importantly, the databases we utilized share structural similarities with those of other regions in Spain and Europe. Therefore, our findings are highly relevant at both national and international levels, and our patient catch-up methodology could be replicated and automated to improve vaccination strategies for various high-risk populations worldwide.

## 5. Conclusions

We present a methodology to identify and catch-up patients under HR-IMD conditions that can be applied to other vaccines and populations. In our retrospective study, including 24 years of follow-up, we showed very low meningococcal vaccination coverage for all risk groups. We identified 1638 patients that required MenB vaccination and 2109 that required MenACWY vaccination using a reliable code algorithm through R software. This study could serve to optimize and automate the catch-up of at-risk patients and improve vaccination coverage.

## Figures and Tables

**Table 1:** High-risk groups for invasive meningococcal disease (IMD) and International Classification of Diseases (ICD) codes used for patient identification.

Risk Group Label	Risk Group Description	ICD-9 Diagnose	ICD-9 Procedure	ICD-10 Diagnose	ICD-10 Procedure	Total
		Count	Count	Count	Count	Count
Asplenia	Individuals with anatomical asplenia or severe splenic dysfunction (e.g., sickle cell anemia) and those scheduled for surgical splenectomy.	13	2	45	5	65
Properdin-complement deficiency	Individuals with properdin deficiency or complement deficiencies.	2	0	2	0	4
Previous IMD	Individuals who have experienced an episode of IMD, regardless of vaccination status prior to the episode.	14	0	18	0	32
Hematopoietic stem cell transplantation	Individuals who have undergone hematopoietic stem cell transplantation	8	9	13	68	98
Outbreak	In outbreak situations where the health authority determines the need for vaccination	0	0	0	0	0
Laboratory personnel	Laboratory personnel (lab technicians and microbiologists) working with samples that may potentially contain *Neisseria meningitidis*	0	0	0	0	0
PLWH	People living with HIV	3	0	11	0	14
Total	-	40	11	89	73	214

**Table 2:** Age and sex of the patients according to high-risk conditions for meningococcal vaccination.

High-Risk Group	No. of Conditions (%)	Males		Females		*p*-Value ^1^
		*n* (%)	Median Age (IRQ)	*n* (%)	Median Age (IRQ)	
Asplenia	855 (31.4%)	432 (50.5%)	58.5 (46.4–69.9)	423 (49.5%)	63.2 (49.2–74.0)	**0.006**
Properdin-complement deficiency	36 (1.3%)	16 (44.4%)	55.5 (46.0–64.7)	20 (55.5%)	47.7 (40.0–57.2)	0.275
Previous IMD	121 (4.4%)	65 (53.7%)	25.5 (19.9–36.2)	56 (46.3%)	26.0 (18.3–43.6)	0.541
HSCT	743 (27.3%)	406 (54.6%)	56.5 (41.7–68.3)	337 (45.4%)	56.3 (39.3–66.4)	0.117
PLWH	955 (35.0%)	711 (74.5%)	55.6 (45.0–62.3)	243 (25.4%)	53.7 (44.7–60.1)	0.090
Total	2710 (100.0%)	1630 (60.1%)	56.1 (43.1–64.7)	1079 (39.8%)	56.7 (42.8–67.1)	-

HSCT, hematopoietic stem cell transplantation; IMD, invasive meningococcal disease; PLWH, people living with HIV. ^1^ Mann–Whitney test was performed (except for properdin-complement deficiency, where *t*-test was performed).

**Table 3:** Meningococcal vaccine doses in each high-risk group, and data stratified by sex.

Risk Group	*n* Total	Vaccine	0 Doses	1 Dose	2 Doses	3 Doses	4 Doses	Males ≥ 1 Dose	Females ≥ 1 Dose	*p*-Value ^1^
Asplenia	855	MenB	516 (60.4%)	22 (2.6%)	300 (35.1%)	13 (1.5%)	4 (0.5%)	150 (34.7%)	189 (44.7%)	<0.001
		MenACWY	615 (71.9%)	63 (7.4%)	174 (20.4%)	1 (0.1%)	2 (0.2%)	98 (22.7%)	142 (33.6%)	<0.001
Properdin-complement deficiency	36	MenB	34 (94.4%)	1 (2.8%)	0 (0.0%)	1 (2.8%)	0 (0.0%)	2 (12.5%)	0 (0.0%)	0.190 ^1^
		MenACWY	33 (91.7%)	2 (5.6%)	1 (2.8%)	0 (0.0%)	0 (0.0%)	2 (12.5%)	1 (5.0%)	0.574 ^1^
Previous IMD	121	MenB	101 (83.5%)	0 (0.0%)	19 (15.7%)	1 (0.8%)	0 (0.0%)	12 (18.5%)	8 (14.3%)	0.537
		MenACWY	82 (67.8%)	34 (28.1%)	4 (3.3%)	1 (0.8%)	0 (0.0%)	21 (32.3%)	18 (32.1%)	0.984
HSCT	743	MenB	480 (64.6%)	43 (5.8%)	210 (28.3%)	6 (0.8%)	4 (0.5%)	147 (36.2%)	116 (34.4%)	0.612
		MenACWY	451 (60.7%)	88 (11.8%)	200 (26.9%)	2 (0.3%)	2 (0.3%)	161 (39.7%)	131 (38.9%)	0.827
PLWH	955	MenACWY	745 (78.0%)	77 (8.1%)	132 (13.8%)	0 (0.0%)	1 (0.1%)	172 (24.2%)	38 (15.6%)	<0.001
Total	1755	MenB	1131 (64.4%)	66 (3.8%)	529 (30.1%)	21 (1.2%)	8 (0.5%)	311 (33.8%)	313 (37.4%)	0.116
	2710	MenACWY	1926 (71.1%)	264 (9.7%)	511 (18.9%)	4 (0.1%)	5 (0.2%)	454 (27.9%)	330 (30.6%)	0.125

HSCT, hematopoietic stem cell transplantation; IMD, invasive meningococcal disease; PLWH, people living with HIV. ^1^ Chi-square test (except for properdin-complement deficiency, where Fisher exact test was calculated).

**Table 4:** Assessment of meningococcal vaccine status and required recommendations.

Assessment	Vaccine Status	MenB, *n* (%)	MenACWY, *n* (%)	Required Recommendations
Full schedule	Adequately vaccinated	18 (1.0%)	378 (13.9%)	None. Correct vaccination status at the time of the analysis
Recently initiated schedule	Adequately vaccinated	8 (0.5%)	0 (0.0%)	
Time interval between primary vaccination schedule and booster dose not exceeded	Adequately vaccinated	0 (0.0%	176 (6.5%)	
Time interval between booster dose and periodic booster dose not exceeded	Adequately vaccinated	96 (5.4%)	0 (0.0%)	
Time interval between primary vaccination doses not exceeded	Adequately vaccinated	10 (0.6%)	62 (2.3%)	
Exceeded recommended interval between schedule and booster dose	Not adequately vaccinated	435 (24.6%)	1 (0.0%)	Book an appointment to continue with vaccination schedule
Exceeded recommended interval between booster dose and periodic booster dose	Not adequately vaccinated	2 (0.1%)	0 (0.0%)	
Exceeded recommended interval between primary vaccination doses	Not adequately vaccinated	56 (3.2%)	171 (6.3%)	
Vaccination not initiated or not documented	Not adequately vaccinated	1145 (64.7%)	1937 (71.1%)	Book an appointment to initiate vaccination schedule

**Table 5:** Vaccine non-coverage by risk group according to the start of the high-risk condition (before or after immunization guideline).

High-Risk Group	*n* Total	Vaccine	Before Immunization Guideline		After Immunization Guideline		*p*-Value ^1^
			*n*	Non-Coverage, *n* (%)	*n*	Non-Coverage, *n* (%)	
Asplenia	855	MenB	386	327 (84.7%)	469	189 (40.3%)	<0.001
		MenACWY	542	473 (87.3%)	313	142 (45.4%)	<0.001
Properdin-complement deficiency	36	MenB	23	22 (95.7%)	13	12 (92.3%)	1.000
		MenACWY	28	26 (92.9%)	8	7 (87.5%)	0.541
Previous IMD	121	MenB	101	91 (90.1%)	20	10 (50.0%)	<0.001
		MenACWY	111	78 (70.3%)	10	4 (40.0%)	0.107
HSCT	743	MenB	460	381 (82.8%)	283	99 (35.0%)	<0.001
		MenACWY	460	359 (78.0%)	283	92 (32.5%)	<0.001
PLWH	955	MenACWY	322	266 (82.6%)	633	479 (75.7%)	0.018

HSCT, hematopoietic stem cell transplantation; IMD, invasive meningococcal disease; PLWH, people living with HIV. ^1^ Chi-square test.

## Data Availability

Data cannot be made publicly available. Those interested in obtaining this information should contact CMBD’s management.

## Abbreviations

The following abbreviations are used in this manuscript:

ATC	Anatomical Therapeutic Chemical Classification System
BDU	User Database (from the Andalusian Health Service)
CMBD	Minimum Basic Data Set
ICD	International Classification of Diseases
IMD	Invasive meningococcal disease
MenACWY	Vaccines against meningococcal serogroups A, C, W, and Y
MenB	Vaccines against meningococcal serogroup B
HSCT	Hematopoietic stem cell transplantation
NUHSA	Unique Health Record Number (from the Andalusian Health Service)
PLWH	People living with HIV

## Appendix A

**Table A1:** Vaccine dosage MenB and MenACWY vaccines in high-risk groups for IMD.

Vaccine Group	Risk Group	Vaccine	Age 1st Dose	Recommended Specific Schedule
MenB	- Asplenia ^1^ - Properdin-complement deficiency ^1^ - Complement inhibitor treatment ^1^ - HSCT ^1^ - Laboratory personnel - IMD episode (2 doses) - Outbreak (2 doses)	4MenB (Bexsero^®^)	2 to 5 months	Primary vaccination schedule: Two doses in the primary series. The first dose should not be administered before 2 months of age. Minimum interval between doses: 2 months. Booster dose: One booster dose between 12 and 15 months of age, with a minimum interval of 6 months from the primary series.
			6 to 11 months	Primary vaccination schedule: Two doses in the primary series, with a minimum interval of 2 months between doses. Booster dose: One booster dose in the second year of life, with a minimum interval of 2 months from the primary series.
			12 to 23 months	Primary vaccination schedule: Two doses in the primary series, with a minimum interval of 2 months between doses. Booster dose: One booster dose with an interval of 12 to 23 months from the primary series.
			Over 2 years	Primary vaccination schedule: Two doses with a minimum interval of 1 month. Booster dose: One booster dose 1 year after completing the primary series.
		FHbp (Trumenba^®^)	Over 10 years	Primary vaccination schedule: Two doses with a minimum interval of 6 months.
MenACWY	- Asplenia ^2^ - Properdin deficiency ^2^ - Complement inhibitor treatment ^2^ - HSCT - PLWH (HIV infection) - Laboratory personnel (1 dose) - IMD episode (1 dose) - Outbreak - (1 dose) - International travelers (1 dose)	MenACWY-TT (Nimenrix^®^)	1.5 to 5 months	Primary vaccination schedule: Two doses in the primary series. Minimum interval between doses: 2 months. Booster dose: One booster dose at 12 months of age, with a minimum interval of 6 months from the primary series.
			6 to 11 months	Primary vaccination schedule: Two doses in the primary series. Minimum interval between doses: 2 months. Second dose at 12 months of age.
			Over 1 year	Primary vaccination schedule: Two doses with a minimum interval of 2 months.
		MenACWY-TT (MenQuadfi^®^)	Over 1 year	Primary vaccination schedule: Two doses with a minimum interval of 2 months.
		MenACWY-CRM (Menveo^®^)	Over 2 years	Primary vaccination schedule: Two doses with a minimum interval of 2 months.

^1^ In individuals with asplenia, properdin and complement deficiencies, complement inhibitor treatment, laboratory personnel, and hematopoietic stem cell transplantation: MenB vaccine require a periodic booster dose every 5 years. ^2^ In individuals with asplenia, properdin and complement deficiencies, and complement inhibitor treatment: MenACWY vaccine requires a periodic booster dose every 5 years.
